# CT based automatic clinical target volume delineation using a dense-fully connected convolution network for cervical Cancer radiation therapy

**DOI:** 10.1186/s12885-020-07595-6

**Published:** 2021-03-08

**Authors:** Zhongjian Ju, Wen Guo, Shanshan Gu, Jin Zhou, Wei Yang, Xiaohu Cong, Xiangkun Dai, Hong Quan, Jie Liu, Baolin Qu, Guocai Liu

**Affiliations:** 1 Department of Radiation Oncology, The First Medical Center, People’s Liberation Army General Hospital, No. 28 Fuxing Road, Haidian District, Beijing, 100853 China; 2 School of Physics Science and Technology, Wuhan University, No. 299, Bayi Road, Luojiashan Street, Wuhan, 430072 China; 3 Department of Magnetic Resonance Imaging, the First Affiliated Hospital of Zhengzhou University, No. 1 Jianshe East Road, Zhengzhou, 450003 China; 4 Beijing Eastraycloud Technology Inc. Chengdu R&D Center.Suite, 1405-1406,Building Guannan Shangyu,NO.1,Xingguang Road,Wuhou District, Chengdu, 610094 China; 5 College of Electrical and Information Engineering, Hunan University, Changsha, 410082 China

**Keywords:** Dense V-net, Convolutional neural network, Automatic delineation, Cervical Cancer, Clinical target volume

## Abstract

**Background:**

It is very important to accurately delineate the CTV on the patient’s three-dimensional CT image in the radiotherapy process. Limited to the scarcity of clinical samples and the difficulty of automatic delineation, the research of automatic delineation of cervical cancer CTV based on CT images for new patients is slow. This study aimed to assess the value of Dense-Fully Connected Convolution Network (Dense V-Net) in predicting Clinical Target Volume (CTV) pre-delineation in cervical cancer patients for radiotherapy.

**Methods:**

In this study, we used Dense V-Net, a dense and fully connected convolutional network with suitable feature learning in small samples to automatically pre-delineate the CTV of cervical cancer patients based on computed tomography (CT) images and then we assessed the outcome. The CT data of 133 patients with stage IB and IIA postoperative cervical cancer with a comparable delineation scope was enrolled in this study. One hundred and thirteen patients were randomly designated as the training set to adjust the model parameters. Twenty cases were used as the test set to assess the network performance. The 8 most representative parameters were also used to assess the pre-sketching accuracy from 3 aspects: sketching similarity, sketching offset, and sketching volume difference.

**Results:**

The results presented that the DSC, DC/mm, HD/cm, MAD/mm, ∆V, SI, IncI and JD of CTV were 0.82 ± 0.03, 4.28 ± 2.35, 1.86 ± 0.48, 2.52 ± 0.40, 0.09 ± 0.05, 0.84 ± 0.04, 0.80 ± 0.05, and 0.30 ± 0.04, respectively, and the results were greater than those with a single network.

**Conclusions:**

Dense V-Net can correctly predict CTV pre-delineation of cervical cancer patients and can be applied in clinical practice after completing simple modifications.

## Background

Cervical cancer is the second most common cancer in Chinese women and ranks fourth for both incidence and mortality in women worldwide, and its morbidity and mortality have revealed an upward inclination in recent years [1]. Radiotherapy is an important treatment for prolonging the life of cervical cancer patients. Radical radiotherapy is suitable for patients with stage I and stage II and whose physical conditions are not suitable for surgery. Adjuvant radiotherapy is effective for patients with intermediate and high risk factors found in postoperative pathological examination. The precise delineation of CTV plays an important role in the radiotherapy process, which is also very important for the design, assessment, and optimization of radiotherapy planning and directly impacts patients’ prognosis. Currently, the work of delineating CTV is mainly done manually by radiotherapists based on three-dimensional CT images. This is time-consuming, labor-intensive, and very boring. Many researchers try to use new methods, such as k-nearest neighbor [2], ATLAS [3], PET-ATLAAS [4], to achieve automatic delineation.

Recently, the use of deep learning for automatic lesion identification has attracted much attention and significant progress has been made in nasopharyngeal carcinoma [5, 6], rectal cancer [7], and other diseases [8, 9]. The CTV area of cervical cancer includes not only imaging visible lesions, but also subclinical lesion areas and possible invasion areas. Even in the same stage, the range of invasion and organ filling are different, which will form individual differences, which will affect the range of delineation. As a result, the research progress in this field is slow, and no relevant results have been reported in previous studies.

Therefore, in this study, we used Dense V-Net to delineate cervical cancer CTV based on CT images automatically to assess the value of Dense V-Net in predicting CTV pre-delineation inpatients with cervical cancer.

## Methods

### Data collection

From May 2016 to June 2019, data were collected from patients with postoperative cervical cancer that were admitted to the Department of Radiotherapy in the First Medical Center of the Chinese People’s Liberation Army General Hospital. This study was conducted according to the Declaration of Helsinki and the ethics committee of our hospital approved this study. All patients signed informed consent.

According to Radiation Therapy Oncology Group (RTOG) CTV consensus definition [10], the CTV range comprises of the uterine body, whole vagina, cervical lesions, bilateral uterine parasites, as well as common iliac, internal iliac, external iliac obturator, and presacral lymphatic drainage regions. The CT images of all patients were obtained using the SIMENS SOMATOM Definition AS large-aperture CT machine. The scanning range was from the top of the liver to the lower end of the perineum. The number of layers was 85–120 and the layer thickness was 5 mm. The scanning parameters were 120KV tube voltage, 400mAs tube current, and the reconstructed voxel value was 512*512* k. During the CT scan, the patient was setup in a supine position and fixed with a thermoplastic phantom.

### Dense V-net for segmentation

**Fig. 1:**
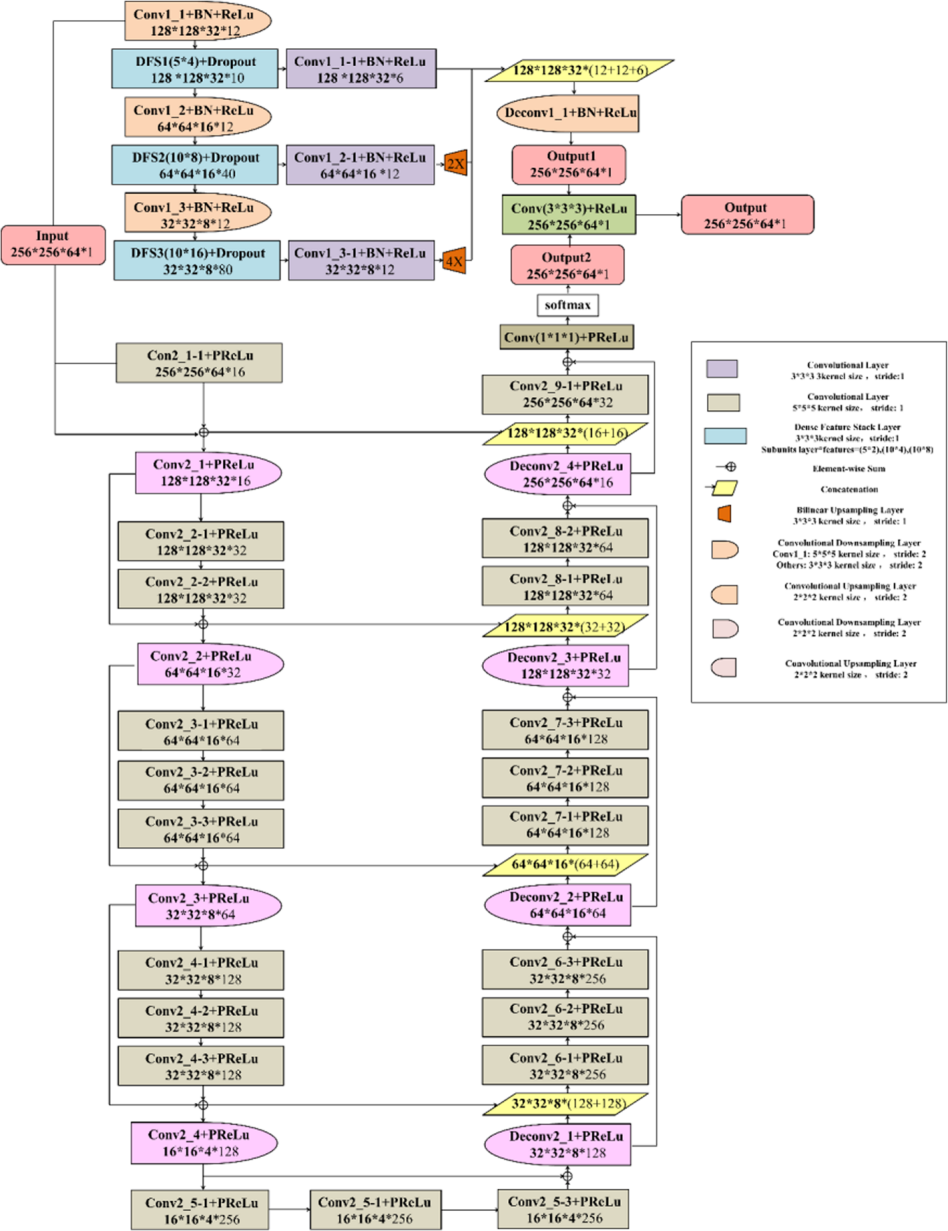
Dense V-Net structure diagram

Dense V-Net [11] is a deep learning network that integrates two deep learning models of Dense Net [12, 13] and V-Net [14, 15]. The structure is shown in Fig. 1. Its three main features are the use of dense connection, horizontal connection, and fusion convolution. Firstly, the input of the dense connection convolution x_l_ layer contains all the outputs of the previous x_0_, x_1_, … …, x_l-1_ layers. Each layer of the network can directly approach the feature maps of the previous layers, involving the loss function and the gradient of the original input, which assists in deepening the network structure and improves the utilization rate of the image features of each layer. The use of dropout clarifies the image information taken for each dense connection and the suitable training outcome can be achieved by using fewer parameters without relearning unnecessary feature mapping between the layers. The use of BN (Batch Normalization) also reduces the over-fitting of the training set with fewer data. Secondly, the horizontal connection joins the convolutional layer through a kernel with assize of 5*5*5 voxels and convolution up-sampling and down-sampling layer with 2*2*2 voxel-wide kernels applied with stride 2. This operation can achieve the effect of improving the output image details, deepening the image contour, and shortening the network fusion time while correctly predicting the image structure. The residual connections are used between convolution operations to break network symmetry and enhance the sensitivity of gradient calculations. In the final prediction, a convolution kernel with a volume of 1 × 1 × 1 is used to perform the convolution operation, the soft-max layer uses the Dice coefficient as the new objective function, and finally outputs the probabilistic segmentation image of the foreground and background regions. Thirdly, the convolution kernel of 3*3*3 is used in the fusion convolutional layer and the rectified linear unit (ReLu) is applied as the activation function so that the image features are fully utilized, and the segmentation outcome is enhanced. Finally, the output of the same size as the original input image is obtained [16].

The fusion network includes the benefits of two single networks. The structure of Dense Net ensures that each layer of the image has the maximum information flow so that the parameters can be used by all the following layers, therefore, improving the utilization rate of image features and quickening the convergence of the target function while reducing unnecessary information. Performing convolution and concatenation operations on three resolution images facilitate multi-scale features extraction and add more details and global data to the results. The structure of V-Net confirms that the output prediction can reserve more image details and maintain the correctness of the image prediction in the case of a small training set. When the network deepens, the results of a deep network are still controlled by the expanding receptive field, which has a significant result in reducing over-fitting.

### The experimental methods

Since the CT image contains a large amount of vacant background and the research focus area is located in the middle of the space, the CT images should be preprocessed. First, the cross-section of the CT image is intercepted at the center of the 320*320 size and resampled by bilinear interpolation to decrease the resolution of the entire training sample to 256*256*k. This operation enhances the proportion of effective data in a single training data, reduces the volume of the sample, and achieves the purpose of enhancing training precision and reducing training time.

Then, a data enhancement process should be performed by sampling and rotating the training samples: we randomly extracted 64 consecutive layers of CT images of each case to attain 10–20 training samples and then we rotated the samples at random angles within ±10 degrees along the x, y, and z axes, respectively, to further expand the data capacity. These two operations can increase the sample size while producing more extensive and accurate training results.

**Fig. 2:**
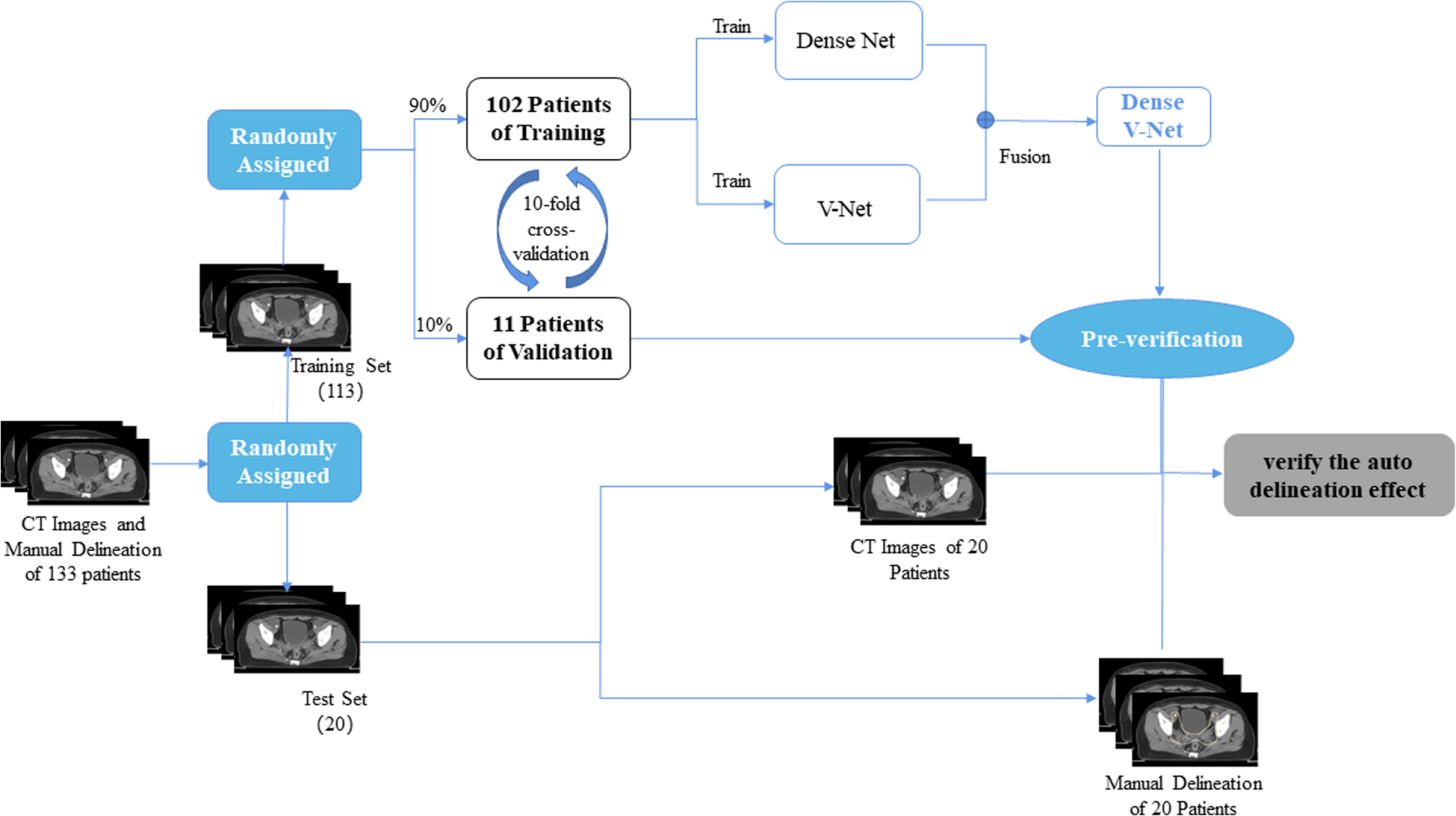
Network training and sketching verification flow chart

Finally, we completed the network training and verification. The details of the flow are shown in Fig. 2. During training, the parameters of V-Net and Dense Net were separately trained and optimized. When the loss function of the two was optimal, the fusion layer was fine-tuned so that Dense V-Net could achieve the best network fusion effect in the shortest time.

Data training, assessment, and testing tasks were all run on servers with dual NVIDIA (GTX 1080) graphics cards. The algorithms used were based on the TensorFlow system architecture and written and tuned in Python. We also used the DSC (Dice Similarity Coefficient) value as the loss function. The initial learning efficiency was set to 0.0005, the learning rate attenuation factor was 0.5, the attenuation step size was 1000, and the number of iterations was set to 10,000.

### The quantitative evaluation

The overall assessment of the automatic delineation was completed using the DSC [17]. To describe the details further, the other seven most representative parameters were used to evaluate the automatic sketching accuracy. They were the three parameters for measuring the degree of contour deviation of the two sketches: deviation of Centroid (DC), Hausdorff Distance (HD) [18], and Minimum Average Distance (MAD), as well as four parameters that measured the difference in volume between the two sketches, namely the Deviation of Volume (∆V), Sensitivity Index (SI), Inclusiveness Index (Incl) [19], and Jaccard Distance (JD) [20]. The image and training results were then transmitted into MIM. Maestro 6.6.5 software to acquire the sketch information and the assessment parameters of the two sketches were calculated on the basis of this platform.

### Statistical analysis

We used the software program SPSS 20.0 (IBM, Chicago, USA) to conduct the statistical analysis. The continuous variables of normal distribution were expressed as mean ± standard deviation, the continuous variables of a non-normal distribution were expressed as median (interquartile range [IQR]), and the categorical variables were expressed as frequency (percentage [%]). For multiple comparisons, each value was compared by one-way ANOVA following a Dunnett test when each datum conformed to a normal distribution while the non-normally distributed continuous data were compared using non-parametric tests. The counting data were tested by a chi-square test. A value of *P* < 0.05 was considered statistically significant.

## Results

### The general characteristics

**Fig. 3:**
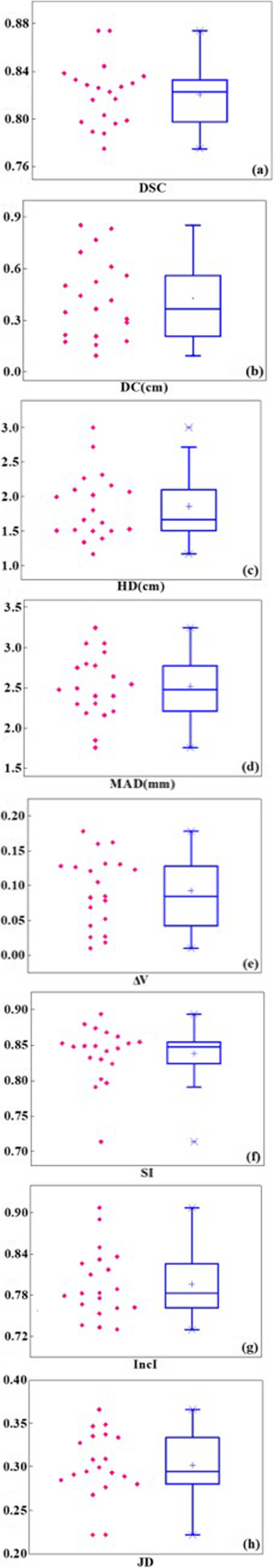
8 parameters scatter box diagram

A total of 133 patients with stage IB and stage IIA, according to 2018 International Federation of Gynecology and Obstetrics (FIGO 2018 stage) Cancer Report [21], were enrolled in this study. The CT images of all the patients were manually delineated by 2 attending physicians and then studied and approved by a senior chief physician. One hundred and thirteen of the patients were randomly selected as the training set and the remaining twenty patients’ CT images were used as the test set to assess the automatic delineation performance of the network. The statistical results of 8 parameters of the CTV automatically delineated by Dense V-Net are shown in Table 1 and the scatter box diagram is shown in Fig. 3.

**Table 1:** Dense V-Net automatically draws parameters

Paraments	DSC	DC/mm	HD/cm	MAD/mm	∆V	SI	IncI	JD
**Minimum**	0.78	0.95	1.17	1.76	0.01	0.71	0.73	0.22
**Maximum**	0.88	8.55	3.01	3.25	0.18	0.89	0.91	0.37
**Median**	0.83	3.92	1.74	2.49	0.10	0.85	0.78	0.30
**Mean**	0.82	4.28	1.86	2.52	0.09	0.84	0.80	0.30
**Standard Deviation**	0.03	2.35	0.48	0.40	0.05	0.04	0.05	0.04

### The DSC score

The results presented that all the 20 cases were higher than the standard 0.75 [22]. Only 5 of them were less than 0.8; the median and mean were both greater than 0.8 and the maximum reached 0.875. This showed that the overall resemblance between the automatic sketch and manual sketch was high.

### The contour deviation

During the completion of the contour deviation, the automatic sketching was stable. The results presented that no extreme point existed, showing that there was no extreme identification error in the automatic delineation; the standard deviation was small, which indicated that the sketch effect was stable. MAD represents the average value of the minimum distance between the two contours and its results were on the order of millimeters, which presented that the inaccurate automatic sketch areas had little effect on the deviation of the two outlines.

### The volume of each orientation

Based on the evaluation of the correctness of the automatic contour orientation, the volume of each orientation was further evaluated by the ∆V, SI, Incl, and JD. ΔV represents the proportion of the volume difference between the two sketches in the manual sketch, which is used to assess the volume stability of the network. The average value was 0.09 and the maximum value was 0.18, suggesting that the automatic sketch was partially or even completely contained. SI and IncI indicated the ratio of the coincidence volume of the two delineations to the manual and automatic contour volume, respectively; the average values were 0.84 and 0.80, respectively. SI was slightly larger than IncI, which meant that the volume of the automatic delineation volume was larger than the manual one overall. The minimum values of the two were 0.71 and 0.73, respectively, and JD represented the complement of the size of the union of the two delineations intersection. The JD values were in a narrow distribution and the standard deviation was 0.04.

### The comparison between the fusion network and a single network

The fusion network was compared with the single network and the results are shown in Table 2. According to the characteristics of each parameter, it was evident that the automatic delineation similarity of the fusion network was significantly higher than the single network. Some representative evaluation parameters, such as DSC, HD, JD, had significant differences in the results (*p* < 0.01),while the other parameters had statistical differences (*p* < 0.05).

**Table 2:** The 8 parameters of network delineation and the single-factor analysis of variance of the single network to the converged network

Paraments	Dense V- Net	Dense Net	P	V-Net	P
**DSC**	0.82 ± 0.03	0.75 ± 0.04	< 0.01	0.76 ± 0.07	< 0.01
**DC/mm**	4.28 ± 2.35	5.17 ± 3.09	< 0.01	4.52 ± 2.35	< 0.01
**HD/cm**	1.86 ± 0.48	2.74 ± 0.99	< 0.01	2.56 ± 0.68	< 0.01
**MAD/mm**	2.52 ± 0.40	2.65 ± 0.58	0.39	2.65 ± 0.30	0.23
**∆V**	0.09 ± 0.05	0.18 ± 0.04	< 0.01	0.12 ± 0.05	0.21
**SI**	0.84 ± 0.04	0.80 ± 0.06	0.05	0.79 ± 0.05	< 0.01
**IncI**	0.80 ± 0.05	0.74 ± 0.07	0.01	0.75 ± 0.09	0.02
**JD**	0.30 ± 0.04	0.41 ± 0.06	< 0.01	0.44 ± 0.05	< 0.01

### The comparison between the automatic delineation among the fusion network and manual delineation

**Fig. 4:**
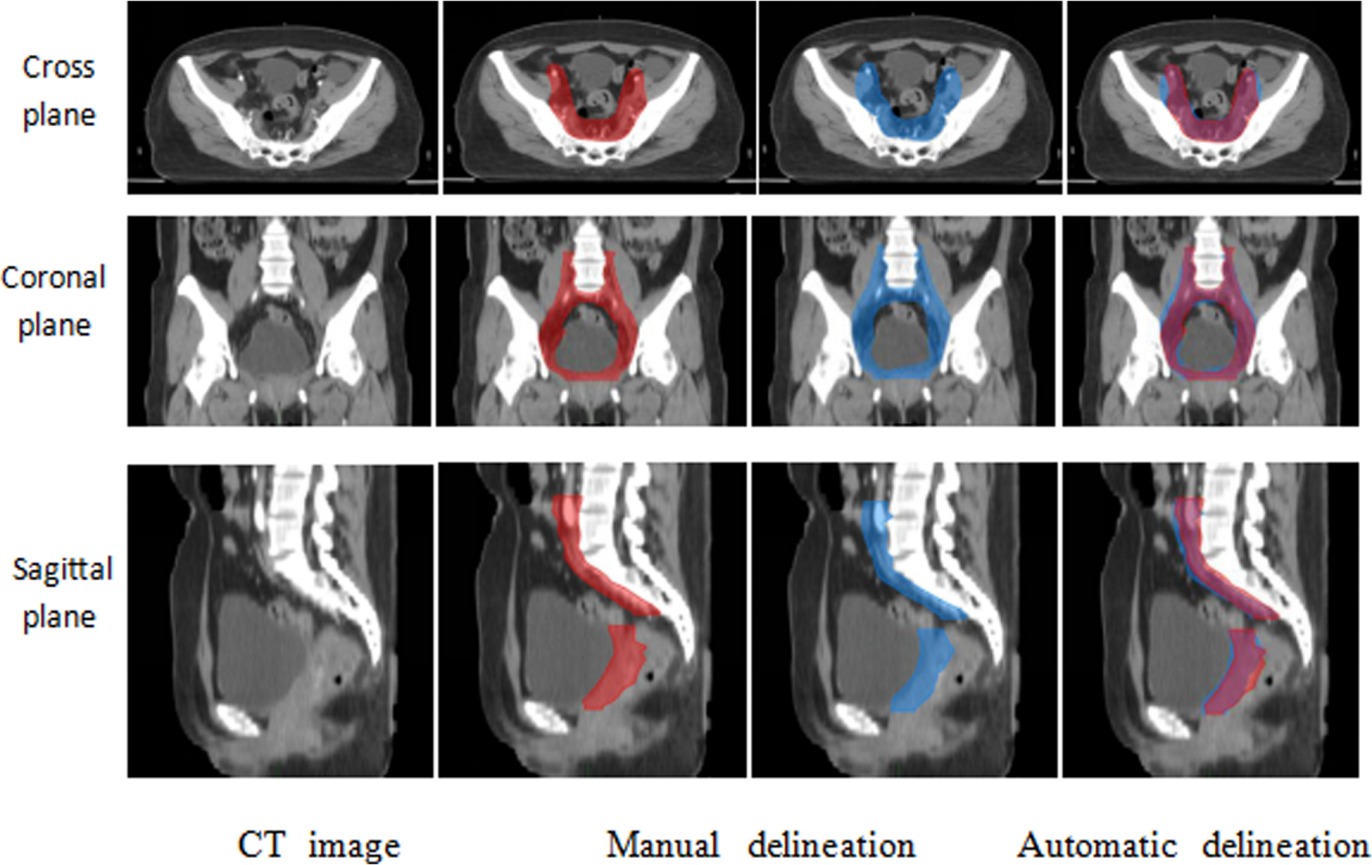
Patient CT image and sketching results

The comparison results of automatic delineation using the fusion network and manual delineation by the radiation oncologist are shown in Fig. 4. The red regions showed the contours of CTV manually drawn by the doctors and the blue regions showed the result of the automatic sketches. It is evident that the results of the two kinds of delineations had a high degree of coincidence.

### The specific segmentation results

**Fig 5:**
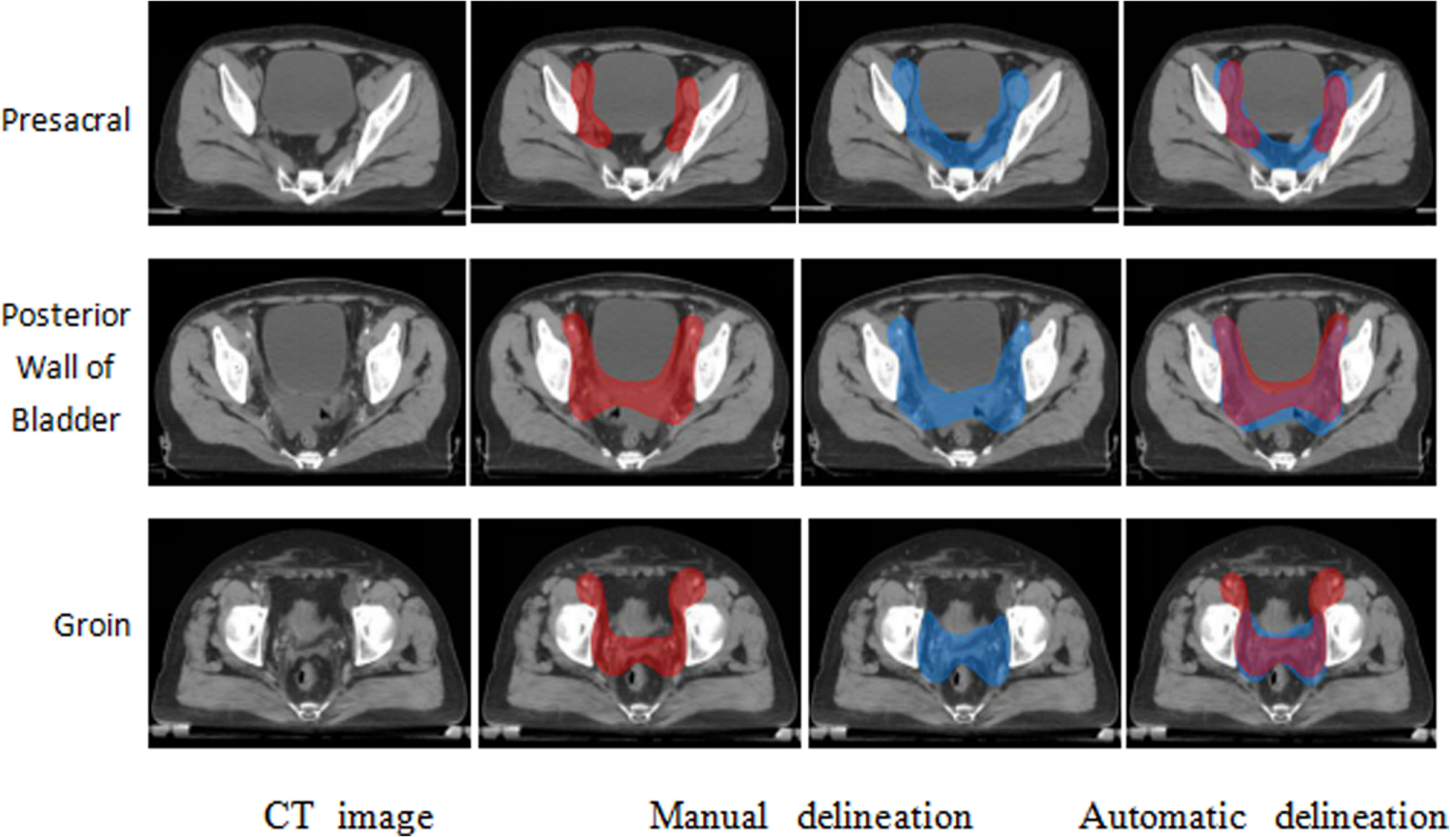
CT cross-sectional images of patients and Comparison of delineations in three conditions of huge difference

A more detailed examination of the segmentation results found that in the following three structures, the network segmentation ability performed poorly. As shown in Fig. 5.

First, at the lower edge of the bifurcation of the presacral lymphatic drainage area, and the upper anterior boundary of the piriformis, the automatic delineation error is generally large. The deep network often fails to accurately identify this boundary, which results in redundant delineation at the anterior edge of the piriformis muscle and the third sacral vertebra.

Secondly, in the area of the posterior wall of the bladder and the anterior wall of the rectum, automatic delineation also has recognition bias. This is mainly due to the fact that when the doctor manually draws it out, the CTV boundary will be extended to the back wall of the bladder for a distance ranging from 1 to 2 mm according to the degree of the patient’s holding back. However, this deep network only pays attention to the significant anatomical differences in this part, and due to the small sample size and unequal extension distance, the learning effect here is poor.

Finally, there are a few cases of vaginal invasion due to disease progression. In actual delineation, doctors often delineate a small number of lymph nodes in the groin area as CTV. This is also difficult for the network to handle.

## Discussion

Medical image analysis, especially medical image segmentation based on deep learning, is one of the most critical areas of current computer vision research. Segmentation is an important processing step in medical images, used for medical scene understanding and image analysis.

The tumor regions segmentation is a research hotspot in medical imaging analysis. There have been many reports on segmentation of visible lesions or GTV (Gross Tumor Volume) and the results show that, the accuracy has surpassed traditional methods, and some results are comparable to expert manual segmentation. For example, Alom [23] automatically outlines the tumor in the lung CT image, and the average segmentation DSC value is 98.32%.

Nevertheless, the identification and delineation of CTV is still very difficult. This is mainly because, in addition to the visible anatomical structure, CTV also includes subclinical potential lesion areas and some lesion invasion areas. These areas are often determined by the doctor’s supervisory judgment and are greatly affected by individual differences, the location of the lesion, and the stage of the cancer. Even if the stage is the same, the scope of tumor invasion and lymphatic involvement are different, which will lead to different delineation scopes. In addition, changes in the patient’s bowel position and bladder filling shape will also affect the recognition and delineation of CTV.

It is of clinical significance to differentiate and delineate CTV in cervical cancer patients. Although there have been some pioneering studies [24–26], they are mainly applied to the self-image sketching of patients, image-guided radiotherapy or the dose superposition evaluation of re-planning, and it is impossible to delineate the CTV of new patients. Due to the scarcity of clinical samples and problems in implementation, the research development of CTV automatic delineation based on CT images of new cervical cancer patients is slow and no relevant results have been reported yet.

To minimize the error of delineation outcomes caused by individual differences in different patients, we used a deep learning method to automatically sketch CTV based on CT images in cervical cancer patients with pelvic radiotherapy. Dense V-Net, a fusion algorithm with an accurate automatic segmentation effect for high deformation soft tissue organs [11],has the advantages of the 2 single algorithms: the dense connection structure is used to understand parameter sharing and strengthen feature transfer; dropout and BN enables a training set with a capacity of 113 to achieve good training results; horizontal connection improves the precision and correctness of the output sketch; and residual connection efficiently solves the gradient disappearance and explosion phenomena generated during training. Its unique fusion convolution layer and the unilateral inhibition of the ReLu function further enhances the outcome of feature learning, quickens the model convergence and improves the sketch efficiency. According to our research results, the characteristics of CTV can be fully learned by Dense V-Net and the automatic delineation outcome is helpful in the case of limited training samples. When designing the radiotherapy plan for cervical cancer, the network can fulfill the automatic pre-sketching of CTV, which greatly improves the clinical work effectiveness.

DSC is used to assess the proportion of the coincidence delineation of 2 sketches in the total outline. When the DSC score is greater than 0.75, it is deliberated that the two regions have a high degree of coincidence [22]. Our study showed that the overall resemblance between the automatic sketch and manual sketch was high.

During the completion of contour deviation, automatic sketching was stable. DC measures the centroid deviation of the two contours, with an average of 4.28 mm, which is less than the scanning layer thickness of 5 mm. HD represents the maximum value of the shortest distance between the two outlines. Statistics demonstrate that no extreme point exists, representing that there is no extreme identification error in automatic delineation; In our experience the standard deviation is small, which specifies that the sketch effect is stable. MAD represents the average value of the minimum distance between the two contours and its results are all on the order of millimeters, which demonstrates that the incorrect automatic sketch areas have little effect on the deviation of the two outlines.

Based on the evaluation of the accuracy of the automatic contour orientation, the volume of each orientation was further evaluated by the ∆V, SI, Incl, and JD.ΔV represents the proportion of the volume difference between the two sketches in the manual sketch, which is used to assess the volume stability of the network. Our study discovered that the volume difference between the two delineations were small and the automatic sketch was partially or even completely contained. SI and IncI indicate the ratio of the coincidence volume of the two delineations to the manual and automatic contour volume, respectively. The results of this study showed that the two delineations had a high degree of coincidence. SI was slightly larger than IncI, which meant that the volume of automatic delineation volume was larger than the manual one overall. The corresponding samples had a large contour deviation degree. JD represents the complement of the size of the union of the two delineations intersection. The JD values were in a narrow distribution, which suggested that the volume offset was small and the network sufficiently identified the sample features.

In this study, the automatic delineation resemblance of the fusion network was significantly higher than that of the single network. Representative evaluation parameters such as DSC, HD, JD, etc. have significant differences in the results while the other parameters had statistical differences. Therefore, automatic sketching using a fusion network has a smaller volume deviation, reduced less range of error identification, lower centroid deviation, and higher volume stability. The difference between the standard deviation also showed that the fusion network had stronger sketching stability and featured an accurate learning ability.

The comparison results of automatic delineation using the fusion network and manual delineation by the radiation oncologists showed that the results of the two kinds of delineations had a high degree of coincidence, which further suggested that Dense V-Net could achieve satisfactory automatic CTV sketching in CT images.

From the analysis of the evaluation parameters, when the DSC value tended to be stable, the reason for the deviation was further assessed by analyzing the other parameters. We found that the network had a weak segmentation ability in some parts of the delineation. It is evident from Fig. 3 (c), (e), and (f) that there were extreme values for HD, ΔV, and SI. The extreme value of HD suggested that there may have been large error identification or local extreme contour deviation. After completing the examination, the reason was identified to be the lack of automatic delineation; on the coronal plane, the upper bound of automatic delineation was much lower than the manual one. The extreme value of ∆V means that the volume of the two sketches was fairly different and we found that the range of the automatic sketch was too large, which essentially included the manual sketch in most areas. The extreme deviation values of SI is due to the insufficient network recognition ability, which results in the overall difference in volume between automatic drawing and manual drawing, and the overlapping volume is small.

However, the network still has many limitations. Firstly, the individual differences in the medical samples were extremely large. Even in the case of a limited sample size, there are a few cases with more specific CTV sketching and the network tends to overlook this abnormal information when learning features, which confines the compatibility of Dense V-Net to various complex conditions. Secondly, this study only focused on the cervical cancer delineation of stage IB and IIA cancer after surgery. Other stages and if surgery or other anti-cancer treatments will affect CTV delineation to a large extent needs further study. Therefore, the effect of automatic delineation for more complex clinical cases needs to be determined. Thirdly, since the judgment of the potential tumor invasion area contained in CTV, particularly the number of lymph nodes that need to be clarified, depends on the clinical experience of radiation oncologists, different doctors have diverse understandings in the delineation of CTV for the same patient, which confines the universality of the network.

## Conclusions

Dense V-Net can accurately predict CTV pre-delineation of cervical cancer patients and can be utilized in clinical practice after simple modifications.

## Data Availability

The datasets used and/or analyzed during the current study available from the corresponding author on reasonable request.

## Abbreviations

CTV: Clinical Target Volume
DSC: Dice Similarity Coefficient
CT: Computed tomography
DSC: Dice Similarity Coefficient
DC: Deviation of Centroid
HD: Hausdorff Distance
MAD: Minimum Average Distance
∆V: Deviation of Volume
SI: Sensitivity Index
JD: Jaccard Distance
IQR: Interquartile range
